# PseudotimeDE-fast: fast testing of differential gene expression along cell pseudotime

**DOI:** 10.1093/bioinformatics/btaf573

**Published:** 2025-10-21

**Authors:** Yuheng Lai, Dongyuan Song, Lucy Xia, Jingyi Jessica Li

**Affiliations:** Department of Statistics, University of Wisconsin-Madison, Madison, WI 53706, United States; Department of Genetics and Genome Sciences, University of Connecticut Health Center, Farmington, CT 06030-6403, United States; Department of ISOM, School of Business and Management, Hong Kong University of Science and Technology, Clear Water Bay, Hong Kong; Department of Statistics and Data Science, University of California, Los Angeles, CA 90095-1554, United States; Biostatistics Program, Fred Hutchinson Cancer Center, Seattle, WA 98109, United States

## Abstract

**Summary:**

Identifying differentially expressed (DE) genes along cell pseudotime is crucial for understanding dynamic biological processes captured by single-cell RNA sequencing. However, existing DE methods either produce invalid *P*-values by ignoring the uncertainty in pseudotime inference or struggle to scale with the growing size of modern datasets. To address these limitations, we introduce PseudotimeDE-fast, a scalable method for detecting DE genes along pseudotime with well-calibrated *P*-values. Through comprehensive simulations and real-data analyses, we demonstrate that PseudotimeDE-fast delivers comparable or superior performance to existing approaches while offering substantial improvements in computational efficiency.

**Availability and implementation:**

PseudotimeDE-fast is implemented in R with Rcpp acceleration and released under the MIT license. The source code is available at: https://github.com/dsong-lab/PseudotimeDE.

## 1 Introduction

Single-cell RNA sequencing (scRNA-seq) technologies have become a powerful tool for uncovering continuous transitions in cell populations. A common approach involves inferring a latent temporal variable, known as “pseudotime,” from gene expression profiles to represent cells’ relative positions along a developmental trajectory (Trapnell *et al.* 2014). To interpret pseudotime, differential expression (DE) analysis is typically performed to identify genes with significant expression changes along the trajectory. Several methods have been developed for this purpose, such as tradeSeq (Van den Berge *et al.* 2020), scMaSigPro (Srivastava *et al.* 2024), and TDEseq (Fan *et al.* 2024). However, these methods rely on regression models that treat pseudotime as fixed, ignoring the uncertainty in its inference. This oversight can lead to invalid *P*-values, as shown in prior studies (Campbell and Yau 2016, Song and Li 2021).

To consider the uncertainty in inferred pseudotime, we previously developed PseudotimeDE (Song and Li 2021), the first DE method to explicitly account for this uncertainty. PseudotimeDE repeatedly performs trajectory (pseudotime) inference on subsampled cells and applies permutations to break the gene expression–pseudotime association, fitting a regression model to generate a null distribution of the test statistic. This approach yields well-calibrated *P*-values and good statistical power. However, its extensive computational demands, due to repeated model fitting on many subsamples, limit its scalability and broader adoption in the single-cell community.

To overcome the computational limitations of PseudotimeDE, we propose PseudotimeDE-fast, a novel method and updated R package for fast testing of gene expression changes along cell pseudotime. Unlike the methods that rely on regression models assuming fixed pseudotime, PseudotimeDE-fast tests the independence between gene expression and pseudotime by treating both as random variables. It implements a hypothesis test using a novel adaptation of the Bergsma–Dassios sign covariance $\tau^{*}$—a robust extension of Kendall’s tau—for sparse data, where $\tau^{*}=0$ if and only if the two variables are independent (Bergsma and Dassios 2014). Through comprehensive simulations and analysis of a large real dataset, we show that PseudotimeDE-fast produces well-calibrated *P*-values, achieves comparable or improved FDR control and power, and is over 100 times faster than existing methods.

## 2 Implementation

PseudotimeDE-fast is implemented in R and can be installed via devtools::install_github(“dsong-lab/PseudotimeDE”). To address the computational bottleneck of its predecessor PseudotimeDE, it replaces the subsampling-and-permutation procedure with a direct, deterministic statistical test. Specifically, it reframes DE analysis as a formal test of independence between the pseudotime vector *X* and the expression vector $Y_{g}$ of gene *g*.

The input consists of a scRNA-seq count matrix $Y=[Y_{1},\ldots ,Y_{p}]\in R^{n\times p}$, where *n* is the number of cells and *p* is the number of genes, and a pseudotime vector $X\in R^{n}$ representing the inferred pseudotime of cells. For each gene g∈{1,…,p}, PseudotimeDE-fast efficiently computes $\tau_{n}^{*}$, a consistent estimator of the Bergsma–Dassios sign covariance $\tau^{*}$:


$$\tau_{n}^{*}(X,Y_{g})=\frac{(n-4)!}{n!}\sum_{\begin{array}{c} 1\leq i,j,k,l\leq n\\ i,j,k,l\;\text{distinct}\; \end{array}}a(X_{i},X_{j},X_{k},X_{l})\cdot a(Y_{gi},Y_{gj},Y_{gk},Y_{gl}),$$

where


$$a(z_{1},z_{2},z_{3},z_{4})=\text{sign}\left(\left|z_{1}-z_{2}\right|+\left|z_{3}-z_{4}\right|-\left|z_{1}-z_{3}\right|-\left|z_{2}-z_{4}\right|\right).$$

The intuition for this measure, a powerful extension of the well-known Kendall’s τ (Kendall 1938), is that it moves beyond comparing simple pairs of points to evaluating all sets of four points (quartets). For each quartet, it checks whether the arrangement of points is “concordant” or “discordant” for both pseudotime and gene expression.

Previously, Heller and Heller (2016) introduced an algorithm to compute $\tau_{n}^{*}$ with $O(n^{2})$ complexity, which becomes computationally prohibitive as *n* (the number of cells) increases. To address this, we developed an optimized algorithm that reduces the complexity of its core step to O(Mn), where M≪n denotes the number of unique expression levels, often small due to sparsity in scRNA-seq data. Details are provided in Supplementary Material 1, available as supplementary data at *Bioinformatics* online. Under the null hypothesis of independence between *X* and $Y_{g}$, $\tau_{n}^{*}$ admits a known limiting distribution, enabling efficient hypothesis testing (Nandy *et al.* 2016). Compared to other rank-based independence tests with similar statistical properties (Shi *et al.* 2022), our implementation achieves near-linear scalability for sparse data, while existing methods typically face computational bottlenecks.

## 3 Results

To evaluate the performance of PseudotimeDE-fast in terms of runtime, *P*-value validity, FDR control, and statistical power for detecting DE genes, we conducted simulations across varying numbers of cells (*n*) and used a large-scale real scRNA-seq dataset (Tsukui *et al.* 2024). We compared PseudotimeDE-fast with state-of-the-art trajectory-based DE methods, including PseudotimeDE (Song and Li 2021)—in both its asymptotic (fix) mode, which ignores pseudotime uncertainty and is not recommended, and its subsampling-and-permutation (permute) mode, which is accurate but computationally intensive—as well as tradeSeq (Van den Berge *et al.* 2020) and TDEseq (Fan *et al.* 2024). The details about the implementation and computational resources are described in Supplementary Material 2, available as supplementary data at *Bioinformatics* online.

**Figure 1.**
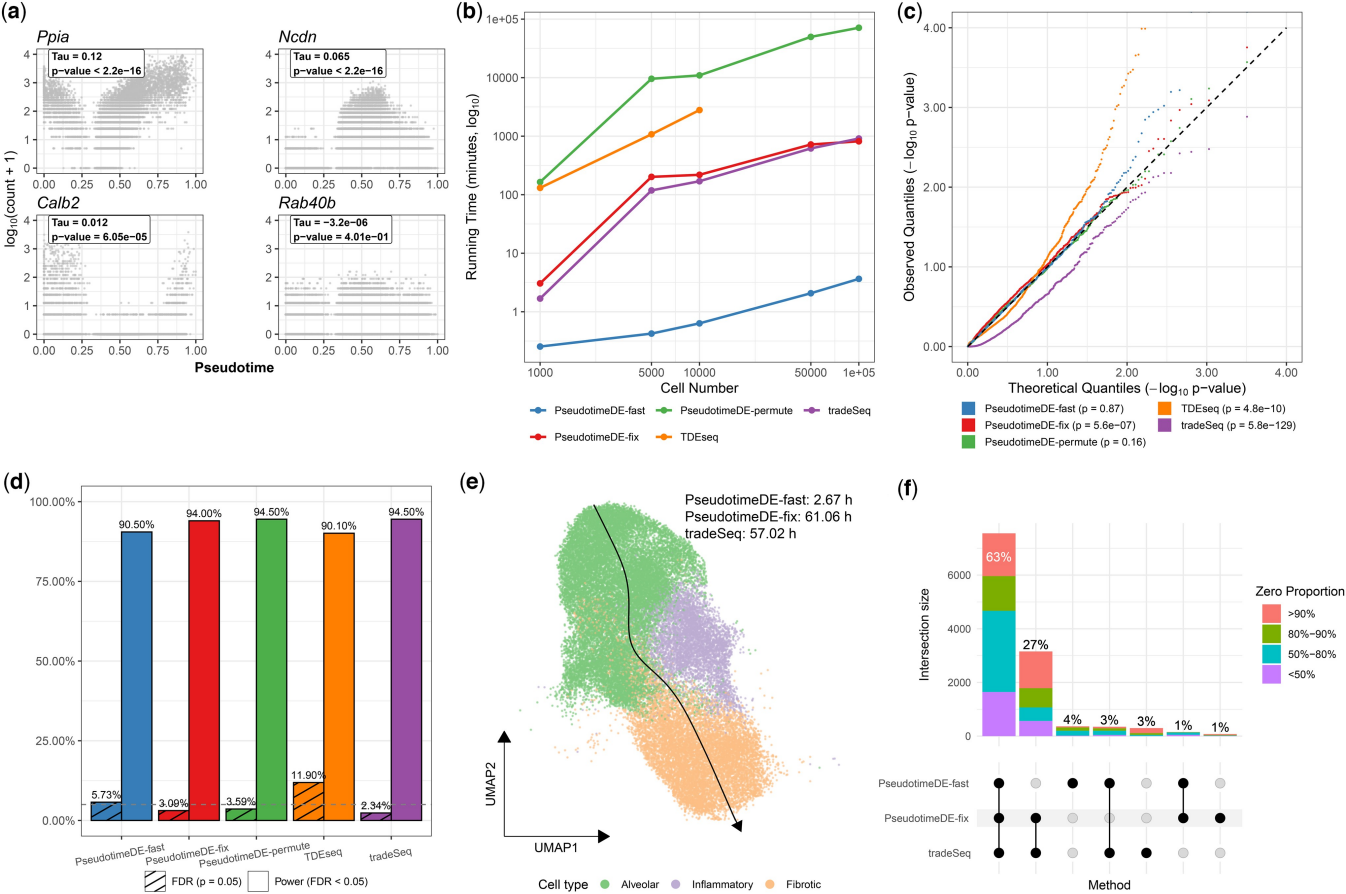
Benchmarking PseudotimeDE-fast against other trajectory-based DE methods. (a) Expression of four example genes along pseudotime. The estimated $\tau_{n}^{*}$ and corresponding *P*-values are shown for each gene. (b) Runtime comparison across different cell numbers (*n*). PseudotimeDE-fast is significantly faster than all other methods. (c) Quantile-quantile plots of *P*-values under the null hypothesis on the $-{\;\text{log}\;}_{10}$ scale. Only PseudotimeDE-fast and PseudotimeDE-permute produce well-calibrated *P*-values, with points falling along the diagonal and Kolmogorov-Smirnov test *P*>.05. (d) FDR and power in simulations. PseudotimeDE-fast achieves reasonable FDR control and comparable power to existing methods. (e) Application to the alveolar fibroblast lineage dataset (Tsukui *et al.* 2024). Cells are visualized by UMAP; colors denote cell types, and the curve indicates the inferred trajectory. PseudotimeDE-fast is 30× faster than PseudotimeDE-fix and tradeSeq. (f) UpSet plot showing overlaps in identified DE genes. PseudotimeDE-fast shares 63% of DE genes with both other methods, indicating high consistency.

We generated synthetic datasets with *p* = 2000 genes (20% DE) and varying numbers of cells *n* ∈ {1000, 5000, 10 000, 50 000, 100 000} using scDesign3 (Song *et al.* 2024), which was trained on a real scRNA-seq dataset of dentate gyrus neurogenesis (Hochgerner *et al.* 2018). Figure 1a shows results for four example genes: PseudotimeDE-fast reported highly significant *P*-values for three DE genes (*Ppia*, *Ncdn*, and *Calb2*) and an insignificant *P*-value for a non-DE gene (*Rab40b*).

Figure 1b compares runtime across methods as *n* increases. All methods support multi-core parallelization, so we set the number of CPUs as 10 for every method. At *n* = 10 000, PseudotimeDE-fast completed in 124.29 s (CPU time): 298 times faster than tradeSeq, 348 times faster than PseudotimeDE-fix, 4408 times faster than TDEseq, and over 24 013 times faster than PseudotimeDE-permute. In terms of clock time, PseudotimeDE-fast finished in just 26.8 s. Note that TDEseq failed to finish within a reasonable runtime (48 h) with *n*=50 000 or more cells (Supplementary Material 2, available as supplementary data at *Bioinformatics* online).

To assess *P*-value validity under the null, we compared *P*-values to the Uniform[0,1] distribution in two ways: (i) quantile-quantile (QQ) plots using $-{\;\text{log}\;}_{10}$  *P*-values, and (ii) Kolmogorov–Smirnov tests using the raw *P*-values (Fig. 1c). PseudotimeDE-fast and PseudotimeDE-permute yielded well-calibrated *P*-values close to the expected uniform distribution. For DE gene detection at *n*=10 000 (additional results in Fig. 1, available as supplementary data at *Bioinformatics* online), PseudotimeDE-fast achieved comparable power and FDR control to state-of-the-art methods while using far less computational time (Fig. 1d). For PseudotimeDE-fix, although its FDR was controlled, its *P*-values showed deviation from the expected uniform distribution (Fig. 1c; Fig. 2, available as supplementary data at *Bioinformatics* online). In addition, although PseudotimeDE-fast showed a slight power loss compared to PseudotimeDE-permute, the few missed genes were highly sparse and often of limited biological interest (Fig. 3, available as supplementary data at *Bioinformatics* online). These results highlight PseudotimeDE-fast as a scalable solution for large-scale pseudotime DE analysis. Note that this simulation has a high signal-to-noise ratio, so pseudotime can be estimated accurately and the “double-dipping” issue (Neufeld *et al.* 2024) is relatively mild. If double-dipping remains a major concern, PseudotimeDE-fast may be combined with the synthetic-null-data approach used by ClusterDE (Song *et al.* 2025) to improve FDR control.

We further evaluated PseudotimeDE-fast using a large-scale scRNA-seq dataset of alveolar fibroblast lineage comprising *n*= 35 096 cells and *p*=12 834 genes (Tsukui *et al.* 2024). This dataset contains a single trajectory, and pseudotime was inferred using Slingshot (Street *et al.* 2018). We applied PseudotimeDE-fast, PseudotimeDE-fix, and tradeSeq, which are the only feasible methods for this dataset, and excluded PseudotimeDE-permute and TDEseq due to scalability issues. PseudotimeDE-fast completed the analysis in under 3 h, making it over 30 times faster than the other two methods, each of which required more than two days (Fig. 1e).

Since ground-truth DE genes are unknown, we assessed consistency across methods as a proxy for power. PseudotimeDE-fast identified a largely overlapping set of DE genes, sharing 63% with both other methods (Fig. 1f). Among DE genes missed by PseudotimeDE-fast but detected by both other methods (27%), 66.1% had zero expression in over 80% of cells, indicating high sparsity and limited informativeness. These results highlight that PseudotimeDE-fast offers substantial speed gains while maintaining good statistical power to existing approaches.

## 4 Discussion

Based on the Bergsma–Dassios sign covariance (an association measure for two random variables), PseudotimeDE-fast does not natively adjust for covariates such as batch effects or sequencing depth; users should therefore correct for confounders prior to analysis. Extending PseudotimeDE-fast to handle covariates would require a conditional (or partial) form of the Bergsma–Dassios sign covariance, which, to our knowledge, has not yet been developed and represents an interesting direction for future research.

## Supplementary Material

btaf573_Supplementary_Data

## Data Availability

The alveolar dataset analyzed in this study is available in the NCBI Gene Expression Omnibus (GEO) under accession GSM6428697 (https://www.ncbi.nlm.nih.gov/geo/query/acc.cgi?acc=GSM6428697).
