# Supplementary material for: PseudotimeDE-fast: fast testing of differential gene expression along cell pseudotime
Source: Bioinformatics. 2025 Oct 21;41(11):btaf573. doi: 10.1093/bioinformatics/btaf573 (PMC12619992; doi:10.1093/bioinformatics/btaf573)
Supplement: btaf573_Supplementary_Data [file btaf573_supplementary_data.pdf]

Supplementary Material

PseudotimeDE-fast: fast and rigorous testing of differential gene expression along cell pseudotime

Yuheng Lai, Dongyuan Song, Lucy Xia, and Jingyi Jessica Li

S1 Algorithm

This section outlines the optimized algorithm for computing the Bergsma–Dassios correlation coefficient (τ_n^*) implemented in PseudotimeDE-fast. The algorithm builds upon the method by Heller and Heller (2016) and incorporates new optimization techniques to improve scalability for large single-cell datasets.

S1.1 Heller's Original Algorithm

We first briefly review the algorithm proposed by Heller and Heller (2016).

S1.1.1 Notation

To facilitate the exposition, we define the following concepts and notation:

- Let $X = (x_1, \ldots, x_n)$ be the inferred pseudotime for each cell and $Y = (y_1, \ldots, y_n)$ be the expression count of a given gene.
- Consider a quartet $\{(x_1, y_1), \ldots, (x_4, y_4)\} \subset \mathbb{R}^2$ and let S_4 be the set of all permutations of the indices of the quartet. The quartet is **concordant** if there exists a permutation $(i, j, k, l) \in S_4$ such that:

$$(x_i, x_j < x_k, x_l) \;\&\; \left[(y_i, y_j < y_k, y_l) \vee (y_i, y_j > y_k, y_l)\right],$$

  where & and $\vee$ denote logical AND and OR, respectively, $(z_1, z_2 < z_3, z_4)$ is shorthand for $\max(z_1, z_2) < \min(z_3, z_4)$, and $(z_1, z_2 > z_3, z_4)$ is shorthand for $\min(z_1, z_2) > \max(z_3, z_4)$.
- The quartet is **discordant** if:

$$\left[(x_i, x_j < x_k, x_l) \vee (x_i, x_j > x_k, x_l)\right] \;\&\; \left[(y_i, y_k < y_j, y_l) \vee (y_i, y_k > y_j, y_l)\right].$$

By Lemma 1 in (Weihs et al., 2016), the estimator τ_n^* can be written in the form of:

$$\tau_n^* = \frac{(n-4)!}{n!}\left(16 \cdot N_c - 8 \cdot N_d\right),$$

where $N_c = |\{(i, j, k, l) : 1 \le i < j < k < l \le n, \{(x_i, y_i), (x_j, y_j), (x_k, y_k), (x_l, y_l)\} \text{ is concordant}\}|$ and $N_d = |\{(i, j, k, l) : 1 \le i < j < k < l \le n, \{(x_i, y_i), (x_j, y_j), (x_k, y_k), (x_l, y_l)\} \text{ is discordant}\}|$ are the number of concordant and discordant quartets respectively.

S1.1.2 Computing N_c and N_d

The counts of concordant quartets (N_c) can be computed through the following formulas:

$$N_c = \sum_{3 \le k \le n-1} \sum_{k < l \le n} \left(\binom{M_<(k, l)}{2} + \binom{M_>(k, l)}{2} \right),$$

where:

$$M_<(k,l) = \left|\{i : x_i < x_k,\ y_i < \min(y_k, y_l)\}\right|,$$
$$M_>(k,l) = \left|\{i : x_i < x_k,\ y_i > \max(y_k, y_l)\}\right|.$$

The quantities $M_<(k,l)$ and $M_>(k,l)$ can be computed in $O(1)$ time using a cumulative distribution matrix $A(r,s)$:

$$A(r,s) = \sum_{i=1}^{n} I(\text{rank}(x_i) \leq r \text{ and } \text{rank}(y_i) \leq s), \tag{S1}$$

where I is the indicator function. Then:

$$M_<(k,l) = A(\text{rank}(x_k) - 1, \text{rank}(\min(y_k, y_l)) - 1),$$
$$M_>(k,l) = \text{rank}(x_k) - A(\text{rank}(x_k), \text{rank}(\max(y_k, y_l))).$$

The computation of N_d is slightly more involved but similarly based on $O(n^2)$ times accessing a matrix, which was detailed in Heller and Heller (2016).

S1.2 Optimized Algorithm in `PseudotimeDE-fast`

As we see, the original algorithm contains an $O(n^2)$ step of computing N_c and N_d, which becomes infeasible for large n. We propose the following optimization for computing N_c and N_d, which substantially improves runtime:

- **Rank-based sparse iteration:** Observations are grouped by unique gene expression ranks, allowing the algorithm to loop only over these distinct ranks instead of all n data points. This eliminates redundant computations for tied values and reduces the $N_c(N_d)$-computing step from $O(n^2)$ to $O(Mn)$, where M is the number of unique expression ranks.

S1.2.1 Implementation Outline

1. **Rank assignment:** Assign ranks to X and Y, treating ties in a dense way. For example, for an input vector $X = (4, 2, 2, 3, 2, 3)$, rank of $X = (3, 1, 1, 2, 1, 2)$.
2. **Matrix construction:** Compute the rank-based matrices (e.g., $A(r,s)$) for computing N_c and N_d, as done in the original algorithm.
3. **Ordering:** Sort the rank pairs $(\text{rank}(x_1), \text{rank}(y_1)), \ldots, (\text{rank}(x_n), \text{rank}(y_n))$ to $(\text{rank}(x_{\sigma(1)}), \text{rank}(y_{\sigma(1)})), \ldots, (\text{rank}(x_{\sigma(n)}), \text{rank}(y_{\sigma(n)}))$ such that $\text{rank}(x_{\sigma(1)}) \leq \ldots \leq \text{rank}(x_{\sigma(n)})$.
4. ***Computing N_c and N_d:** Group data by unique ranks and iterate only through unique ranks, compute counts of concordant/discordant quartets using the matrices.

S1.3 Pseudocode

Algorithm 1 provides a pseudocode implementation of the optimized τ_n^* computation. Optimized steps are highlighted in red.

Algorithm 1 Optimized τ_n^* Computation

Require: X: Vector of pseudotime values, $X = (x_1, x_2, \ldots, x_n)$; Y: Vector of the expression count of a given gene, $Y = (y_1, y_2, \ldots, y_n)$

Rank Assignment:
Compute dense ranks for X and Y to handle ties
$X_{\text{rank}} \leftarrow$ ranks of X
$Y_{\text{rank}} \leftarrow$ ranks of Y
Cumulative Count Matrix Construction:
Build matrix $A(r, s)$ as described in S1
Build $U(r, s)$ for computing N_d as in Heller and Heller (2016) (top of page 5)
Ordering:
Sort the rank pairs $(X_{\text{rank}}, Y_{\text{rank}})$ by ascending order of X_{rank}
Computing of N_c and N_d:
Initialize $numCon \leftarrow 0$, $numDis \leftarrow 0$
Precompute Y_{rank} frequencies:
$Y_{\text{count}}[r] \leftarrow$ number of observations with $Y_{\text{rank}} = r$ // avoid recomputing counts in inner loop
for each i from 1 to $n - 1$ **do**
  Decrement $Y_{\text{count}}[Y_{\text{rank}}[i]]$ by 1 // maintain remaining counts
  for each r from 1 to $\max(Y_{\text{rank}})$ **do**
    // iterate only over unique ranks, not all points
    // original iteration: **for** each j from $i + 1$ to n **do**
    // $r = Y_{\text{rank}}[j]$
    Retrieve $numCon_{i,r} \leftarrow$ increment of N_c, $numDis_{i,r} \leftarrow$ increment of N_d from $A(\cdot, \cdot)$ and $U(\cdot, \cdot)$ based on current ranks $X_{\text{rank}}[i], Y_{\text{rank}}[i], r$ // $O(1)$ from precomputed matrix
    Update $numCon \leftarrow numCon + (Y_{\text{count}}[r] \times numCon_{i,r})$, $numDis \leftarrow numDis + (Y_{\text{count}}[r] \times numDis_{i,r})$ // avoid redundant counting
  end for
end for
return $\dfrac{16 \times numCon - 8 \times numDis}{n(n-1)(n-2)(n-3)}$

S2 Implementation

We used R version 4.4.0 (2024-04-24) on the Ubuntu 20.04.6 LTS system, which had an AMD EPYC 7H12, 2.6GHz, 64-CPU Core, and 3.25 TB Memory.

For pseudotime inference, we used the R package `slingshot` (version 2.12.0). We followed the tutorial `https://bioconductor.org/packages/devel/bioc/vignettes/slingshot/inst/doc/vignette.html` to run slingshot for inferring a single lineage.

For pseudotime-related DE analysis, we compared with R package `PseudotiomeDE` (version 1.0.1), `tradeSeq` (version 1.13.06), and `TDEseq` (version 1.1).

For visualization, we used the R package `ggplot2` (version 3.5.1) and `ggpubr` (version 0.6.0).

S3 Datasets

This section provides details on the generation of simulated datasets and the sources of real data.

S3.1 Simulation

We generated synthetic single-cell RNA sequencing (scRNA-seq) datasets comprising 2,000 genes, with 20% designated as differentially expressed (DE), and varying sample sizes of $\{1{,}000, 5{,}000, 10{,}000, 50{,}000, 100{,}000\}$ cells. The simulations were conducted using `scDesign3` (Song et al., 2024), leveraging a model fitted to a real scRNA-seq dataset from the dentate gyrus neurogenesis

1. **Pseudotime Inference** Principal Component Analysis (PCA) was performed on the real scRNA-seq data using the `irlba` package for dimension reduction, retaining the first two principal components. Trajectory inference was conducted using the `slingshot` package to assign pseudotime values to each cell, which were subsequently rescaled.
2. **Data Construction:** The `construct_data()` function from `scDesign3` was used to prepare the dataset, setting the desired number of simulated cells and including relevant covariates such as pseudotime.
3. **Marginal Distribution Fitting:** Using `fit_marginal()`, we modeled the mean gene expression along pseudotime with spline-based regression, assuming a Negative Binomial (NB) distribution to capture variability in count data.
4. **Differential Expression (DE) Assignment:** Subsequently, 20% of the genes were randomly designated as DE. For non-DE genes, their mean expression values were set to remain constant across pseudotime, effectively simulating genes without differential expression.
5. **Copula Model Fitting:** The `fit_copula()` function applied a Gaussian copula to model dependencies between gene expressions, ensuring realistic correlation structures in the simulated data. Further, we selectively preserved dependencies among DE genes while enforcing independence among non-DE genes, aligning with the assumption that only DE genes are involved along the pseudotime trajectory.
6. **Count Data Simulation:** The `simu_new()` function generated new count matrices based on the fitted marginal and copula models, incorporating the assigned DE genes. This resulted in simulated datasets that reflect both DE and non-DE gene expression patterns.

S4 Figures

S4.1 Real data

We used the dataset studying the alveolar fibroblast lineage (Tsukui et al., 2024). The dataset contains $12,834$ genes, $35,096$ cells, and three cell types. The pseudotime was inferred by `slingshot` (Street et al., 2018) with code from `https://github.com/TatsuyaTsukui/AlveolarLineage`.

References

Heller, Y. and Heller, R. (2016). Computing the bergsma dassios sign-covariance. *arXiv preprint arXiv:1605.08732*.

Song, D., Wang, Q., Yan, G., Liu, T., Sun, T., and Li, J. J. (2024). scdesign3 generates realistic in silico data for multimodal single-cell and spatial omics. *Nature Biotechnology*, 42(2):247–252.

Street, K., Risso, D., Fletcher, R. B., Das, D., Ngai, J., Yosef, N., Purdom, E., and Dudoit, S. (2018). Slingshot: cell lineage and pseudotime inference for single-cell transcriptomics. *BMC Genomics*, 19:1–16.

Tsukui, T., Wolters, P. J., and Sheppard, D. (2024). Alveolar fibroblast lineage orchestrates lung inflammation and fibrosis. *Nature*, 631(8021):627–634.

Weihs, L., Drton, M., and Leung, D. (2016). Efficient computation of the bergsma-dassios sign covariance. *Computational Statistics*, 31:315–328.

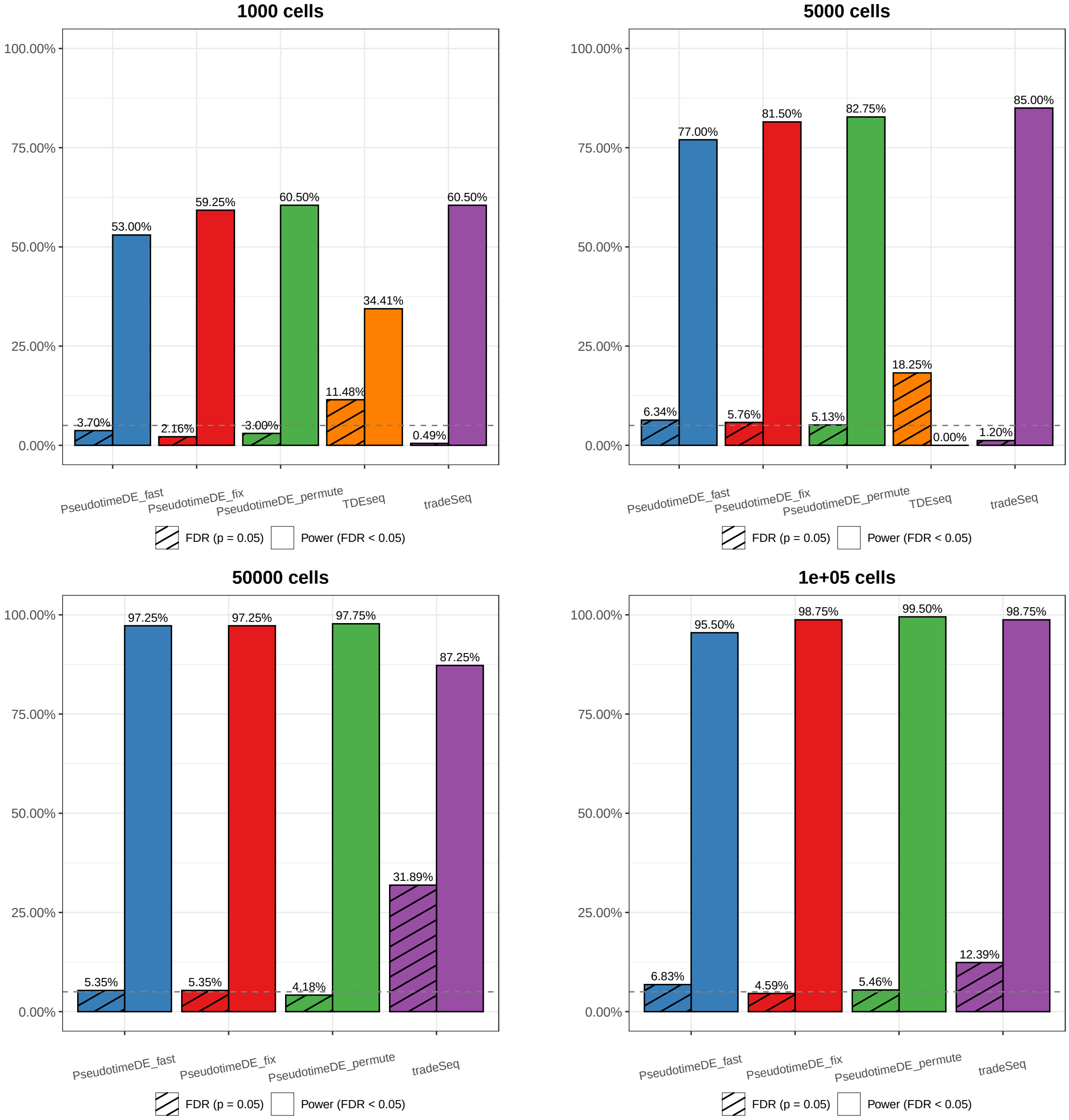

Figure S1: FDR and power of PseudotimeDE-fast against other methods under different numbers of cells.

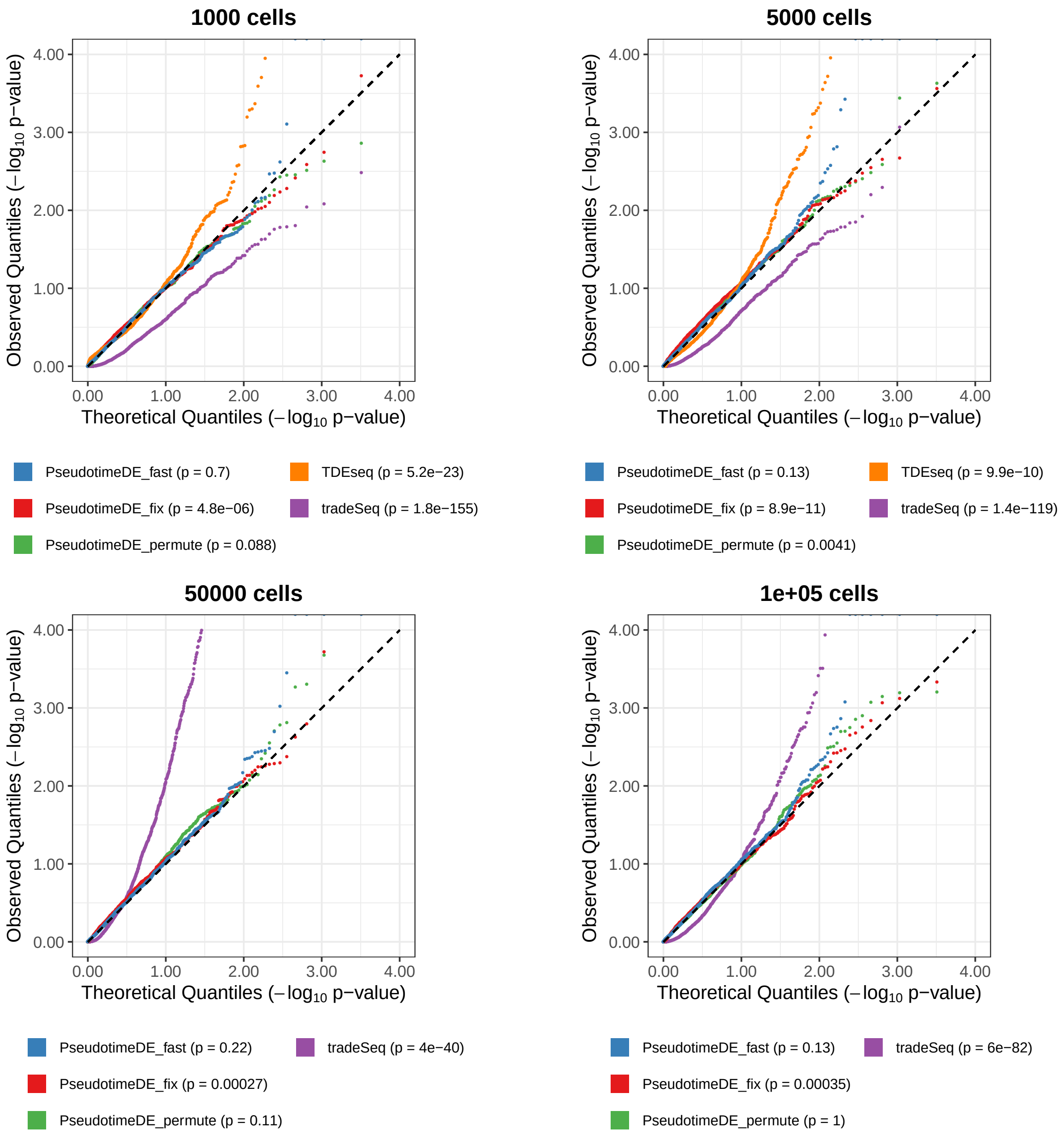

Figure S2: Quantile-quantile (QQ) plot of p-values from PseudotimeDE-fast against other methods under different numbers of cells.

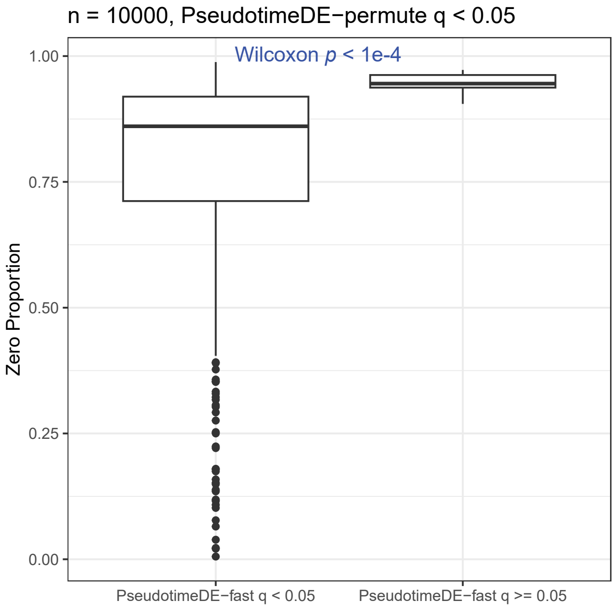

Figure S3: Exploring the zero proportions of genes identified or missed by PseudotimeDE-fast compared to PseudotimeDE-permute.
